# Epi-STEP: A multidisciplinary transition model for patients with epilepsy

**DOI:** 10.1016/j.mex.2026.103914

**Published:** 2026-04-14

**Authors:** Valentina Giobbe, Giorgia Mulè, Martina Paola Zanaboni, Elena Ballante, Natascia Brondino, Massimiliano Celario, Francesca Ferraro, Domenico Lomonaco, Lia Macina, Martina Maria Mensi, Isabella Orlando, Ludovica Pasca, Elena Tartara, Costanza Varesio, Valentina De Giorgis

**Affiliations:** a Department of Brain and Behavioral Sciences, University of Pavia, Pavia, Italy; b Department of Child Neurology and Psychiatry, IRCCS Mondino Foundation, Full Member of ERN-EpiCARE, Pavia, Italy; c Department of Political and Social Sciences, University of Pavia, 27100 Pavia, Italy; d Department of Mental Health and Addiction, ASST Pavia, 27100 Pavia, Italy; e Epilepsy Center, IRCCS Mondino Foundation, Pavia, Italy

**Keywords:** Epilepsy, Transition, Adolescent, Patient perspective, Caregiver perspective, Quality of life, Unmet needs, Protocol

## Abstract

By involving a multidisciplinary team, the model creates a holistic care pathway that supports the medical, psychological, and social needs of adolescents and their caregivers during the transition process. Epilepsy is a prevalent neurological disorder in childhood, and many childhood-onset epilepsy cases persist into adulthood, necessitating a transition from pediatric to adult care. This transition requires a structured process to address medical and psychosocial l needs; however, many epilepsy centers lack formal programs to support this transition, and there is a need for a standardized approach to ensure that patients and their families are adequately prepared. Therefore, we developed the Epi-STEP protocol, a multidisciplinary transition model for young adults with epilepsy and their caregivers. This monocentric observational study will assess the potential usefulness of the transition process for patients with epilepsy aged 16 and older, recruited from the Child Neuropsychiatry Unit at the IRCCS Mondino Foundation. Data will be collected at three time points: at the last visit at the Child Neuropsychiatry Unit (T0), at the first visit at the Adult Epileptology Service (T1), and at a 12-month follow-up (T2). Assessment tools will include both standardized instruments and ad-hoc questionnaires specifically developed to comprehensively evaluate the transition process and will be completed by patients, caregivers, and healthcare professionals. This study will evaluate the utilityof the transition process, assessing readiness for adult care and psychosocial well-being of the patients involved. Findings will guide targeted interventions to improve clinical outcomes, enhance transition readiness, and provide better support for patients and families during this critical phase. Finally, this model designed for epilepsy could be further adapted to other chronic conditions requiring structured transition regimens.

**Trial registration:**

The study has been registered at ClinicalTrials.gov (registration number NCT06778772) on the 16th January 2025.

## Graphical abstract

**Figure:**
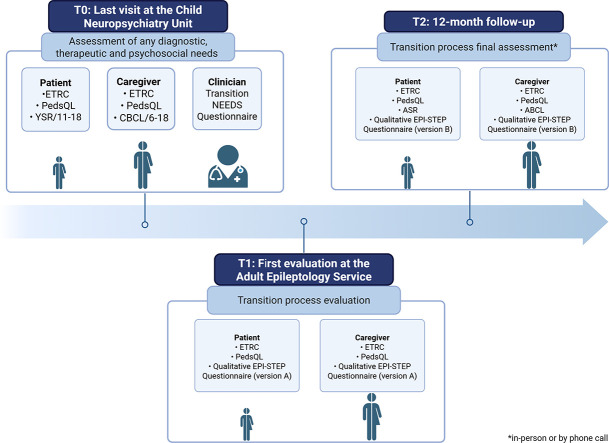

**Specifications table**

**Table:** 

**Subject area**	*Neuroscience*
**More specific subject area**	Medicine, psychology
**Name of your protocol**	Epi-STEP: A Multidisciplinary Transition Model for Patients with Epilepsy
**Reagents/tools**	Not applicable
**Experimental design**	Monocentric observational study with an approximal duration of 48 months. Data will be collected at three time points: on the last visit at the Child Neuropsychiatry Unit (T0), on the first evaluation at the Adult Epileptology Service (T1) and on a 12-month follow-up appointment (T2).
**Trial registration**	The study has been registered at ClinicalTrials.gov (registration number NCT06778772) on the 16th January 2025.
**Ethics**	Prior participation to the study, informed consent will be obtained by patients and by their caregivers. If patients are under 18 years old, consent will be obtained by their legal guardian.
**Value of the Protocol**	By involving a multidisciplinary team, the model creates a holistic care pathway that supports the medical, psychological, and social needs of adolescents and their caregivers during the transition process. This model designed for epilepsy could be further adapted to other chronic conditions requiring structured transition regimens

## Background

Epilepsy is a neurological disease characterized by an enduring predisposition to generate epileptic seizures and often carries neurobiological, cognitive, psychological, and social consequences [1]. It is a common neurological condition in childhood, with an incidence of 5–7 cases per 10,000 children and adolescents [2]. Many childhood-onset epilepsy cases do not achieve remission in adulthood [3], thus necessitating a transfer from pediatric to adult epileptology [4]. This transfer, however, goes beyond a simple "handover" of care; it involves a well-coordinated process addressing the medical, psychosocial, and psychoeducational needs of patients with epilepsy (PWE) and their families [5].

Transition is a structured process that supports adolescents and young adults with chronic conditions in moving from child-centered to adult-oriented healthcare systems, ensuring continuity of care while addressing medical and psychosocial needs. Unlike the transfer of care, which represents a single event, transition is a gradual and dynamic process requiring preparation, patient engagement, and coordination between healthcare providers. The primary aim of the transition process is to minimise the risk of clinical deterioration, suboptimal treatment adherence, and loss to follow-up, all of which may substantially compromise the quality of life of patients and their families [6]. Well-organized transition pathways have been associated with improved long-term outcomes and better patient engagement. When inadequately managed, this phase may lead to poor adherence and loss to follow-up, with potential negative consequences on clinical outcomes [5].

Despite the necessity of a holistic transition process has been recognized, it is still challenging to apply and maintain effective transition programs, and it is only in recent times that shared operational models have emerged as viable solutions [[7], [8], [9], [10]]. Currently, many specialized epilepsy centers lack structured programs designed to support a transition process that adequately addresses the diverse needs of both PWEs and their families. Often, pediatric and adult care teams work independently, making collaboration difficult: while pediatric care emphasizes developmental and psychosocial factors with holistic, family-centered approach, adult care tends to focus more on disease management, with an emphasis on clinical treatments. This shift can pose significant challenges for those with psychiatric comorbidities, cognitive impairments, or specialized care requirements [5].

At the Pediatric and Adolescent Epilepsy Center of IRCCS Mondino Foundation (Pavia, Italy), 15-18% patients are over the age of 14 and present complex, drug-resistant epilepsy, often accompanied by neuropsychiatric comorbidities. To address these needs, we developed an integrated transition service that fosters collaboration between pediatric and adult neurologists. To further systematize this approach, we developed EPI-STEP (Support, Transition, Education, and Pathways for patients with EPIlepsy), a structured and replicable transition program organized into three main phases: (T0) multidisciplinary assessment at the Child Neuropsychiatry Unit, (T1) joint transition visit with pediatric and adult care teams, and (T2) follow-up after transfer to adult services. The program is supported by standardized assessment tools and the involvement of a dedicated transition coordinator, while allowing care to be tailored to the individual clinical, psychological, and social needs of each patient.The EPI-STEP model, validated within a transition protocol approved by the local Ethics Committee, provides a structured framework to support the transition of PWE from pediatric to adult care. The primary objective is to assess transition readiness in patients and their caregivers, reflecting their preparedness to assume responsibility for healthcare management, treatment adherence, and communication with clinicians during the transition process. Secondary objectives include the evaluation of health-related quality of life (HRQoL) and behavioral and emotional functioning, as well as the identification of unmet clinical and psychosocial needs potentially affecting transition readiness. The protocol further explores patient and caregiver experiences of the transition process, including perceived adequacy of information, expectations regarding adult care, and concerns related to continuity and autonomy. Collectively, these measures aim to provide a multidimensional characterization of factors associated with transition readiness and to inform the development of an individualized transition pathway to adult epileptology services.

## Description of protocol

This is a monocentric observational study approved by our local Ethics Committee (approval code 2023–3.11/173) and registered at https://clinicaltrials.gov (code NCT06778772) on 16th January 2025. Prior participation, patients and caregivers will be fully informed about the study procedure and will be required to sign a written informed consent.

### Participants

Participants will be recruited by the Child Neuropsychiatry Unit of the IRCCS Mondino Foundation. Inclusion criteria are as follows: a) being at least 16 years old b) clinical diagnosis of epilepsy of any aetiology, for which a clinical follow-up over time is expected.

Exclusion criteria are: a) Patients who don’t receive the indication to continue the epileptological follow-up after their last visit at the Child Neuropsychiatry Unit; b) caregivers/patients who choose not to continue follow-up care at our center.

#### Sample size calculation

Being the primary outcome purely descriptive, it is not possible to calculate the required sample size. At the Pediatric and Adolescent Epilepsy Center of IRCCS C. Mondino, which follows approximately 900 patients annually, 13% are over the age of 16, satisfying the inclusion criteria. Considering a maximum of 25% of subjects that don’t give the consent to the participation, we planned to enroll a minimum of 90 participants. With the sample planned, considering a percentage of readiness expected of 70%, with a confidence level of 0.95 we can reach a precision of the estimate of 9%.

### Procedures

The estimated total duration of the study is 48 months, of which 36 months will be employed in participants’ recruitment and minimum follow-ups will be organized after 12 months.

To evaluate the effectiveness of the transition process for both PWEs and their caregivers, data will be collected on the last visit at the Child Neuropsychiatry Unit (T0), on the first evaluation at the Adult Epileptology Service (T1) - usually between 6 and 18 months depending on the clinical needs of the PWEs - and on a 12-month follow-up appointment (T2). Study data will be collected in pseudo-anonymised form in an appropriate Case Report Forms (CRF; see Supplementary material 1), designed ad hoc and developed in compliance with good clinical practice (GCP) guidelines.

#### T0- therapeutical, clinical, psychiatric and neuropsychological re-evaluation

The evaluation at the time of the study enrollment (T0) will be conducted either in-patient or in outpatient regimen, based on the clinical complexity of the participant’s condition and certainty of diagnosis.

The diagnostic and therapeutic re-evaluation will be carried out at the Child Neuropsychiatry facility. Specifically, transition package will comprise:

- anamnestic interview.
- therapeutic reassessment.
- re-evaluation of the diagnosis, including EEG, brain MRI, metabolic and genetic tests, if necessary.
- psychodiagnostic and neuropsychological assessment through standardized tests and clinical interviews (for detail please refer to “Measure” paragraph).

The diagnostic framework and therapeutic indications formulated by the pediatric neurologist will be shared with the general practitioner and adult neurologist, through a “Transition Passport” (see Supplementary material 2), in order to update them and to ensure the involvement of these crucial professionals in the follow-up and future care of the patients.

#### T1-First transition appointment

After reassessing the patient's clinical condition and confirming the indication for the transition process, the Epilepsy Nurse (Transition Coordinator) arranges the logistical details of the appointment (T1) at the Adult Neurology Service, ensuring the co-presence of both the pediatric and adult neurologists, and other healthcare practitioners, if needed.

#### T2-Follow-up transition appointment

Around 12 months (T2) after the first transition assessment, the PWEs/caregivers are further interviewed in person or by telephone regarding their transfer experience.

#### Measures

During the last evaluation at the Child Neuropsychiatry Unit (T0), subjects will complete the Italian version of the following tools:

• Patient-Reported Questionnaires:

- Epilepsy Transition Readiness Checklist (ETRC) [11]
- Pediatric Quality of Life Inventory - Teen version (PedsQL 4.0) [12]
- Youth Self-Report (YSR/11–18) [13]

• Caregiver-Reported Questionnaires:

- Child Behavior Checklist (CBCL/6–18) [14]
- Caregiver versions of the ETRC and the PedsQL.

• Pediatric Neurologist-Reported Questionnaire:

- Screening Transition NEEDS Questionnaire (see Supplementary material3)

During appointments at the Adult Epileptology Service (T1 and T2), participants and caregivers will complete assessments as follows:

• T1 Visit:

- Participants and Caregivers: PedsQL, ETRC, and Qualitative EPI-STEP Questionnaire version A (see Supplementary material 4)

• T2 Visit:

- Participants and Caregivers: PedsQL, ETRC, Adult Behavior Checklist (ABCL) [13], Adult Self-Report (ASR) [13] and Qualitative Epi-STEP Questionnaire version B (see Supplementary material 4)

#### Assessment tools overview

**Screening Transition NEEDS Questionnaire** (see Supplementary material 3): to provide a comprehensive understanding of the factors influencing transition readiness and to identify potential barriers, the Screening Transition NEEDS Questionnaire was developed. This tool, grounded in clinical and theoretical literature, offers a systematic and practical method for evaluating the complex needs of patients transitioning from pediatric to adult epilepsy care. The clinician completes five subsequent items assessing five crucial domains in the transition process (diagnostic, therapeutic, social, educational and emotional issues). Each question from A1 to A5 is worth 20% in the case of a negative response (“No”), while a positive response (“Yes”) corresponds to a score of 0%. A higher score indicates a greater degree of readiness. A score of 100% indicates that there are no gaps and so that the patient is fully ready for the transition. The cut-off score on the questionnaire is set at 60%. If a patient scores below this threshold, it suggests that there are critical aspects that need to be addressed before proceeding with the transfer. During the clinical visit, if the clinician identifies significant deficiencies in any domain (scores between 60% and 80%), appropriate interventions or recommendations will be made to address those areas. For instance, if a patient is undergoing psychodiagnostic evaluation due to a psychiatric comorbidity, and this aspect is not yet resolved, the clinician may identify a gap in the psychological/emotional domain and decide to delay the transition. Similarly, if there has been a recent change in the therapeutic regimen that requires follow-up, the therapeutic domain may need further attention before transitioning to adult care. At the end of this evaluation, a final question (question B) assesses whether the clinician believes that the patient and their family are ready for the transfer to adult neurology. Question B has two response options, “Yes” or “No”. The former response represents 100%, indicating readiness for the transition, while the latter corresponds to 0%, meaning that there are gaps that still need to be addressed, resulting in a postponement of the transition.

**Qualitative Epi-STEP Questionnaire** (see Supplementary material 4): explores timing and introduction to the transition process, quality of information provided, expectations from adult neurology care and concerns about continuity of care and autonomy, with parallel patient and caregiver versions. This tool includes two versions: Version A, completed at T1, and Version B, completed at T2. Both assess the same domains, except that Version A is completed at the beginning of the transition process, while Version B is filled when the patient is already under the care of adult services and contains an additional question to assess the overall level of satisfaction with the transition process. This tool, grounded in clinical and theoretical literature, offers a systematic and practical method for evaluating the complex needs of patients transitioning from pediatric to adult epilepsy care.

**YSR/11–18** (Youth Self-Report) and **ASR** (Adult Self-Report): evaluate adaptive functioning, behavioral/emotional problems and social competencies, the former in teenagers, the latter in adults. Since participants are still under 18 at the beginning of the transition process, at T0 they complete the YSR/11–18, while at T2 they fill out the ASR.

**CBCL/6–18** (Child Behavior Checklist) **and ABCL** (Adult Behavior Checklist): caregiver reports providing insight into the individual's behavior and emotional state. The CBCL/6–18 is a parallel version of the YSR/11–18 and is given at T0, the ABCL is the parallel version of the ASR and is handed at T2.

**PedsQL** (Pediatric Quality of Life Inventory): assesses health-related quality of life (HRQoL) across physical, emotional, and social domains, with parallel patient and caregiver versions. According to the patient's age, the appropriate version will be administered, either the Teen (age 13–18) or the Young adult (age 18–25) version [12].

**ETRC** (Epilepsy Transition Readiness Checklist): explores the patient’s readiness for the transition process in 16–17-year-old teenagers, with parallel patient and caregiver versions. This checklist investigates to which extent the patient is capable and ready to manage healthcare issues, compliance to pharmacological therapy and communication with clinicians. These measures provide a structured framework for evaluating transition readiness and identifying barriers to successful transitions from pediatric to adult epilepsy care.

Patients should indicate the response that best applies to them on a 4-point Likert scale (0 = I don't know; 1 = No, but I am learning to do this; 2 = Yes, I have started doing this; 3 = Yes, I always do this), with a total score ranging from 0 to 72. A higher score indicates greater readiness.

For the caregiver version, questions 1 to 16 assess the caregiver's perception of the child's readiness using a 4-point Likert scale (0 =No, my child does not know this, 1= No, but my child is learning to do this, 2= Yes, my child has started doing this, 3= Yes, my child always does this), with a total score of 48. A higher score indicates a greater perception of readiness by the caregiver. For questions 17 to 25, which assess the parent's knowledge regarding the transition, the responses are based on a 4-point Likert scale (0 = No, I do not know 1 = I know some of this 2 = I know most of this 3 = I know all about this), with a total score ranging from 0 to 24. Higher scores indicate greater knowledge. The scoring system for the questionnaire, adapted for this study, is essential for assessing participants' readiness in a standardized and objective manner.

### Outcomes

#### Primary outcome measures

To evaluate transition readiness in PWEs and their caregivers, defined as the preparedness to manage healthcare needs, treatment adherence, and communication with clinicians, using the ETRC questionnaire

#### Secondary outcome measures

- To explore perceptions of HRQoL) among PWEs and their families using PedsQL scores.
- To assess behavioral and emotional functioning using YSR/ASR and CBCL/ABCL questionnaires.
- To explore associations between ETRC scores and patients’ clinical characteristics, HRQoL, and comorbidities in relation to transition readiness, and to identify individuals at higher risk of poor transition outcomes who may benefit from targeted social or psychoeducational interventions.

### Statistical analysis

To address the primary outcome, transition readiness (ETRC scores) will be evaluated longitudinally across T0, T1, and T2. The proportion of ready subjects will be reported at each timepoint. Changes in ETRC scores over time will be analyzed using repeated measures ANOVA. Pairwise comparisons between timepoints (T0 vs T1, T0 vs T2, T1 vs T2) will be performed using paired t-tests or non-parametric equivalents where appropriate. To investigate secondary outcomes, changes in HRQoL (PedsQL scores) will be analyzed across T0, T1, and T2. Behavioral and emotional outcomes (YSR/ASR and CBCL/ABCL) will be compared between T0 and T2 using paired t-tests or non-parametric equivalents where appropriate.Associations between ETRC scores and clinical, demographic, and psychosocial variables will be explored using appropriate correlation analyses.The significance level will be set at 5%.

## Protocol validation

Epilepsy is a chronic neurological disorder often associated with psychological, cognitive and social difficulties [1]. Even in cases of seizure remission, PWEs may face challenges related to social isolation, reduced educational attainment, and economic dependency [15]. Given not only the physical, but also the emotional burden of this condition, nowadays epilepsy treatments focus not only on seizure control, but also on social and psychological aspects that may impact on the overall HRQoL of patients. For this reason, it is essential to develop a well-structured transition process, which should begin in early adolescence and extend through adulthood, that addresses all these clinical needs [16].

By adopting a coordinated multidisciplinary approach, transition protocols should aim at promoting autonomy and providing education and support for both PWEs and families [10].

The Epi-STEP protocol offers a multidisciplinary transition model that aims to provide a smooth and comprehensive transfer from pediatric to adult care. This protocol is designed to assess both the PWE’s and the family’s readiness for transition, with a particular focus on mapping unmet needs that may require ongoing management. By involving a team of specialists— including pediatric neurologists, psychologists, adult neurologists and psychiatrists —the model creates a holistic care pathway that supports the PWE’s medical, psychological, and social needs as they reach adulthood. To evaluate the effectiveness of the transition process, the study utilizes both standardized and created ad-hoc assessment tools that help the clinician to evaluate the readiness of the PWE and their caregiver, as well as their psychosocial well-being and transition management by clinicians.

In conclusion, by integrating a multidisciplinary approach and addressing both medical and psychosocial aspects, the Epi-STEP protocol will help to develop targeted intervention strategies for patients and their families, with the goal of enhancing clinical and psychosocial support during the critical process of transition of PWEs to adulthood and improving the dedicated services. Finally, this model tailored for epilepsy could be as well adapted to other healthcare settings seeking to improve the transition process for individuals with chronic conditions.

## Limitations

None.

**Supplementary material 1**, format .xls, Case Report Form, database to collect study data and instructions on how to fill it.

## Declaration of competing interest

The authors declare the following financial interests/personal relationships which may be considered as potential competing interests:

V.D. received research grants from Jazz Pharmaceuticals, speaker and consultancy fee from Italfarmaco S.p.a, Marinus, Jazz Pharmaceuticals, Nutricia Gmbh, Vitaflo, Dr Schar Kanso; and has served as consultant/advisor for Longboard and Dr Schar Kanso. ET received speakers’ fees from Angelini Pharma, EISAI.

## Data Availability

No data was used for the research described in the article.

## Appendix Supplementary materials

**Supplementary material 2**, format .doc, Transition Passport-Anamnestic Report, diagnostic framework and therapeutic indications formulated by the pediatric neurologist for each participant. Image, application 1

**Supplementary material 3**, format .doc, Screening TRANSITION NEEDS Questionnaire, useful tool completed by a clinician to provide a comprehensive understanding of the factors influencing transition readiness and to identify potential barriers Image, application 2

**Supplementary material 4**, format .doc, Qualitative EPI-STEP Questionnaire (version A and B), explores timing and introduction to the transition process, quality of information provided, expectations from adult neurology care and concerns about continuity of care and autonomy, with parallel patient and caregiver versions. Version A is completed at T1, while Version B is completed at T2. Image, application 3

Image, application 4
