# Supplementary material for: Epi-STEP: A multidisciplinary transition model for patients with epilepsy
Source: MethodsX. 2026 Apr 14;16:103914. doi: 10.1016/j.mex.2026.103914 (PMC13185769; doi:10.1016/j.mex.2026.103914)
Supplement: Supplementary file 2 — Supplementary material 3, format .doc, Screening TRANSITION NEEDS Questionnaire, useful tool completed by a clinician to provide a comprehensive understanding of the factors influencing transition readiness and to identify potential barriers [file mmc2.docx]

# Transition Passport – Anamnestic report

Patient name and surname: _________________________________________________________________________

Patient date of birth: __/__/____

Profession: ______________________________________________________________________________________

Living situation: __________________________________________________________________________________ Today’s date: __/__/____

1. Age at first seizure:__________________________________________________________________________
2. Seizure types:______________________________________________________________________________
3. Epilepsy type:______________________________________________________________________________
4. Etiology:__________________________________________________________________________________
5. Seizure triggers:____________________________________________________________________________
6. History of status epilepticus: ☐ Yes ☐ No

If yes please describe (how many episodes, triggers, treatments, and response):

____________________________________________________________________________________________________________________________________________________________________________________________________________________________________________________________________________________________________________________________________________________________________

1. Seizure frequency in the last year:_______________________________________________________________
2. Last seizure type(s) and date: ____________________________________________________________________________________________________________________________________________________________________________________________________________________________________________________________________________________________________________________________________________________________________
3. Longest period of seizure freedom:_____________________________________________________________
4. EEG history: ____________________________________________________________________________________________________________________________________________________________________________________________________________________________________________________________________________________________________________________________________________________________________
5. Last EEG findings:

____________________________________________________________________________________________________________________________________________________________________________________________________________________________________________________________________________________________________________________________________________________________________

1. Neurological examination: ☐ Normal ☐ Abnormal

If abnormal please describe:

____________________________________________________________________________________________________________________________________________________________________________________________________________________________________________________________________________________________________________________________________________________________________

1. Neurological comorbidities: ☐ Yes ☐ No

If yes please describe: ____________________________________________________________________________________________________________________________________________________________________________________________________________________________________________________________________________________________________________________________________________________________________

1. Neuropsychological assessment: ☐ Yes ☐ No

If yes please describe (date, domains investigated, results/diagnosis):

____________________________________________________________________________________________________________________________________________________________________________________________________________________________________________________________________________________________________________________________________________________________________

1. Psychiatric comorbidities: ☐ Yes ☐ No

If yes please describe: ____________________________________________________________________________________________________________________________________________________________________________________________________________________________________________________________________________________________________________________________________________________________________

1. Psychological support in the past: ☐ Yes ☐ No
2. Ongoing psychological support: ☐ Yes ☐ No
3. Internist comorbidities: ☐ Yes ☐ No

If yes describe: ____________________________________________________________________________________________________________________________________________________________________________________________________________________________________________________________________________________________________________________________________________________________________

1. MRI: ☐ Yes ☐ No

If yes please describe MRI date and findings: ____________________________________________________________________________________________________________________________________________________________________________________________________________________________________________________________________________________________________________________________________________________________________

1. Other radiological exams: ☐ Yes ☐ No

Other radiological exams findings:

____________________________________________________________________________________________________________________________________________________________________________________________________________________________________________________________________________________________________________________________________________________________________

1. Metabolic exams: ☐ Yes ☐ No

Metabolic exams results:

____________________________________________________________________________________________________________________________________________________________________________________________________________________________________________________________________________________________________________________________________________________________________

1. Genetic testing: ☐ Yes ☐ No

Date:____________________________________________________________________________________

Type:____________________________________________________________________________________

Results:____________________________________________________________________________________________________________________________________________________________________________________________________________________________________________________________________

1. ASM ongoing (please describe ASM type, dosage, and tolerability): ___________________________________________________________________________________________________________________________________________________________________________________________________________________________________________________________________________
2. ASM history (please describe ASM type, dosage, and reason for suspension): __________________________________________________________________________________________________________________________________________________________________________________

__________________________________________________________________________________________________________________________________________________________________________________

_________________________________________________________________________________________

1. Other therapies (e.g. KDT, VNS): ☐ Yes ☐ No

If yes please describe: ____________________________________________________________________________________________________________________________________________________________________________________________________________________________________________________________________________________________________________________________________________________________________

1. Other therapies ongoing: ☐ Yes ☐ No

If yes please describe type and dosage: ____________________________________________________________________________________________________________________________________________________________________________________________________________________________________________________________________________________________________________________________________________________________________

1. Epilepsy surgery: ☐ Yes ☐ No

If yes please describe date, surgery type, histological findings: _________________________________________________________________________________________

_________________________________________________________________________________________

_________________________________________________________________________________________

_________________________________________________________________________________________

_________________________________________________________________________________________

1. Seizure freedom after surgery: ☐ Yes ☐ No
2. History of febrile seizures: ☐ Yes ☐ No
3. Pregnancy and birth history: ☐ Normal ☐ Abnormal

If abnormal please describe:

____________________________________________________________________________________________________________________________________________________________________________________________________________________________________________________________________________________________________________________________________________________________________

1. Developmental milestones: ☐ Normal ☐ Abnormal

If abnormal please describe:

____________________________________________________________________________________________________________________________________________________________________________________________________________________________________________________________________________________________________________________________________________________________________

1. Rehabilitation: ☐ Yes ☐ No

If yes please describe rehabilitation type (eg. speech therapy, neuropsychological):

__________________________________________________________________________________________________________________________________________________________________________________

1. Sleep disturbance: ☐ Yes ☐ No

If yes please describe sleep problems (e.g. insomnia, nightmares):

____________________________________________________________________________________________________________________________________________________________________________________________________________________________________________________________________________________________________________________________________________________________________

1. Physical Activity Level: ☐ Active (regular exercise) ☐ Moderately active ☐ Sedentary
2. Dietary Habits: ☐ Normal ☐ Special Diet (e.g., low-sodium)
   If special diet, please describe:

___________________________________________________________________________________________________________________________________________________________________________________________________________________________________________________________________________

1. Substance use (e.g. alcohol, smoking, recreational drugs): ☐ Yes ☐ No

If yes please describe type and frequency

__________________________________________________________________________________________________________________________________________________________________________________

1. Social support: ☐ Strong (family, friends, community) ☐ Moderate ☐ Minimal
2. Family history positive for epilepsy: ☐ Yes ☐ No

If yes please describe: _________________________________________________________________________________________

_________________________________________________________________________________________

_________________________________________________________________________________________

_________________________________________________________________________________________

_________________________________________________________________________________________

1. Family history positive for other neurological conditions: ☐ Yes ☐ No

If yes please describe: _________________________________________________________________________________________

_________________________________________________________________________________________

_________________________________________________________________________________________

_________________________________________________________________________________________

_________________________________________________________________________________________

1. Family history positive for psychiatric conditions: ☐ Yes ☐ No

If yes please describe: _________________________________________________________________________________________

_________________________________________________________________________________________

_________________________________________________________________________________________

_________________________________________________________________________________________

_________________________________________________________________________________________

1. Family history positive for internist conditions: ☐ Yes ☐ No

If yes please describe: _________________________________________________________________________________________

_________________________________________________________________________________________

_________________________________________________________________________________________

_________________________________________________________________________________________

_________________________________________________________________________________________

Abbreviations: ASM, antiseizure medication; KDT, ketogenic dietary therapies: VNS, vagus nerve stimulation
