# Supplementary material for: Epi-STEP: A multidisciplinary transition model for patients with epilepsy
Source: MethodsX. 2026 Apr 14;16:103914. doi: 10.1016/j.mex.2026.103914 (PMC13185769; doi:10.1016/j.mex.2026.103914)
Supplement: Supplementary file 3 — Supplementary material 4, format .doc, Qualitative EPI-STEP Questionnaire (version A and B), explores timing and introduction to the transition process, quality of information provided, expectations from adult neurology care and concerns about continuity of care and autonomy, with parallel patient and caregiver versions. Version A is completed at T1, while Version B is completed at T2. [file mmc3.docx]

**SUPPLEMENTARY MATERIAL 3**

**Screening TRANSITION NEEDS Questionnaire**

*Completed by the Attending Doctor*

The questionnaire helps clinicians identify and address any gaps in the transition process, ensuring that both the patient and their family are fully prepared for the transition. By focusing on the patient's readiness and identifying any deficits in care, social support, education, or emotional well-being, healthcare providers (HCPs) can facilitate a smoother transition and improve long-term outcomes for the patient.

This tool serves as a valuable resource for HCPs, offering a comprehensive yet simple and efficient method for assessing the complex needs of patients with epilepsy, particularly during the transition phase.

Patient's Name: ____________________________
Patient's Date of Birth: **/**/____
Completed by Dr. _____________________________
Today's Date: **/**/____

**A1.** Do you believe that there are any diagnostic aspects still lacking at the time of transition?

- No
- Yes (please specify: __________________________________________)

**A2.** Do you believe that there are any therapeutic aspects still lacking at the time of transition?

- No
- Yes (please specify: __________________________________________)

**A3.** Do you believe that there are any healthcare/social aspects still lacking at the time of transition?

- No
- Yes (please specify: __________________________________________)

**A4.** Do you believe that there are any educational/work aspects still lacking at the time of transition?

- No
- Yes (please specify: __________________________________________)

**A5.** Do you believe that there are any psychological/emotional aspects still lacking at the time of transition?

- No
- Yes (please specify: __________________________________________)

**B.** Do you believe that the patient and their family are ready for the transition to adult Neurology?

- No (please specify: __________________________________________)
- Yes

**Total score: __________**

**Scoring:**

Each question from A1 to A5 is worth 20% in the case of a negative response (“no”), while a positive response (“yes”) corresponds to a score of 0%. A higher score indicates a greater degree of readiness.

A score of 100% indicates that there are no gaps and the patient is fully ready for the transition. The cut-off score on the questionnaire is set at 60%. If a patient scores below this threshold, it suggests that there are critical aspects that need to be addressed before proceeding with the transition.

During the clinical visit, if the clinician identifies significant deficiencies in any domain (scores between 60% and 80%), appropriate interventions or recommendations will be developed to address those areas.

At the end of this evaluation, a final question (question B) assesses whether the clinician believes that the patient and their family are ready for the transition to adult neurology. Question B has two response options, “Yes” or “No”. A positive response corresponds to 100%, indicating readiness for the transition, while "No" represents 0%, meaning that there are gaps that still need to be addressed, resulting in a postponement of the transition (exclusion criteria).
