# Supplementary material for: Epi-STEP: A multidisciplinary transition model for patients with epilepsy
Source: MethodsX. 2026 Apr 14;16:103914. doi: 10.1016/j.mex.2026.103914 (PMC13185769; doi:10.1016/j.mex.2026.103914)
Supplement: Supplementary file 4 [file mmc4.docx]

**SUPPLEMENTARY MATERIAL 4**

**Version A**

**Qualitative EPI-STEP Questionnaire**

***Caregiver version***
**Instructions**: please mark the box that best applies. There are no right or wrong answers. Your responses will remain confidential.

Child's Name: _____________
Child's Date of Birth:____
Today's Date: ____

1. At what age did you first hear about the transition to adult Neurology?

- 16 years or younger
- 17 years
- 18 years
- Never
- Older than 18 years (please specify the age: ____________)

1. How much time passed between when you first heard about the transition and the first visit to adult Neurology?

- Less than a year
- 1 year
- 2 years
- More than 3 years
- I don't remember

1. How do you evaluate the information provided about the transition process?

- Very poor
- Poor
- Sufficient
- Good
- Very good

1. What do you expect from the adult Neurology service's care? (You can select more than one option)

- Finding a more effective treatment
- The same type of care
- Finding a diagnosis
- Improvement in care (please specify in which areas: _________________________)
- Other (please specify: ________________________________________________)

1. What are your concerns regarding the transition to the adult Neurology service?

- Less attention to my child's social, educational, or work-related needs
- Difficulty for my child in scheduling or attending visits independently
- That my child may not feel comfortable with the new doctor
- That the new doctor may not be familiar with my child's condition
- Other (please specify:___________________________________________________)

**Version A**

**Qualitative EPI-STEP Questionnaire**

***Patient version***
**Instructions**: please mark the box that best applies. There are no right or wrong answers. Your responses will remain confidential.

Name: ____________________________
Date of Birth: ____
Today's Date:____

1. At what age did you first hear about the transition to adult Neurology?

- 16 years or younger
- 17 years
- 18 years
- Older than 18 years (please specify the age: ____________)
- Never

1. How much time passed between when you first heard about the transition and the first visit to adult Neurology?

- Less than a year
- 1 year
- 2 years
- More than 3 years
- I don't remember

1. How do you evaluate the information provided about the transition process to adult Neurology?

- Very poor
- Poor
- Sufficient
- Good
- Very good

1. What do you expect from the care provided by the adult Neurology service? (You can select more than one option)

- Finding a more effective treatment
- The same type of care
- Finding a diagnosis
- Improvement in care (please specify in which areas: _________________________)
- Other (please specify: _________________________________________________)

1. What are your concerns regarding the transition to the adult Neurology service?

- Less attention to my social, educational, or work-related needs
- Difficulty in scheduling or attending visits independently
- Not feeling comfortable with the new doctor
- The new doctor may not be familiar with my condition
- Other (please specify: __________________________________________________)

**Version B**

**Qualitative EPI-STEP Questionnaire**

***Caregiver version***
**Instructions**: please mark the box that best applies. There are no right or wrong answers. Your responses will remain confidential.

Child's Name: _____________
Child's Date of Birth:____
Today's Date: ____

1. At what age did your child transition to the adult Neurology service?

- Never
- 16 years or younger
- 17 years
- 18 years
- Older than 18 years (please specify the age: ____________)

1. How much time has passed since your child’s last visit to the adult Neurology service?

- Less than a year
- 1 year
- 2 years
- More than 3 years
- I don't remember

1. How would you evaluate the information provided during the transition process?

- Very poor
- Poor
- Sufficient
- Good
- Very good

1. What do you expect from your child’s follow-up care in the adult Neurology service? (You may select more than one option)

- Finding a more effective treatment
- The same type of care
- Finding a diagnosis
- Improvement in care (please specify in which areas: _________________________)
- Other (please specify: ________________________________________________)

1. What concerns do you have about your child continuing follow-up care in the adult Neurology service?

- Less attention to my child's social, educational, or work-related needs
- Difficulty for my child in scheduling or attending visits independently
- That my child may not feel comfortable with the neurologist
- Concern that the neurologist may not be familiar with my child's condition
- Other (please specify:___________________________________________________)

1. Overall, how would you rate the transition from pediatric services to adult services?

- Very negative
- Negative
- Neutral
- Positive
- Very positive

Version B

**Qualitative EPI-STEP Questionnaire**

***Patient version***
**Instructions**: please mark the box that best applies. There are no right or wrong answers. Your responses will remain confidential.

Name: ____________________________
Date of Birth: ____
Today's Date:____

1. At what age did you transition to the adult Neurology service?

- Never
- 16 years or younger
- 17 years
- 18 years
- Older than 18 years (please specify the age: ____________)

1. How much time has passed since your last visit to the adult Neurology service?

- Less than a year
- 1 year
- 2 years
- More than 3 years
- I don't remember

1. How would you evaluate the information provided during the transition process?

- Very poor
- Poor
- Sufficient
- Good
- Very good

1. What do you expect from the follow-up care in the adult Neurology service? (You may select more than one option)

- Finding a more effective treatment
- The same type of care
- Finding a diagnosis
- Improvement in care (please specify in which areas: _________________________)
- Other (please specify: ________________________________________________)

1. What concerns do you have about continuing follow-up care in the adult Neurology service?

- Less attention to my child's social, educational, or work-related needs
- Difficulty for my child in scheduling or attending visits independently
- That my child may not feel comfortable with the neurologist
- Concern that the neurologist may not be familiar with my child's condition
- Other (please specify:___________________________________________________)

1. Overall, how would you rate the transition from pediatric services to adult services?

- Very negative
- Negative
- Neutral
- Positive
- Very positive
